# CT-based Radiogenomics Framework for COVID-19 Using ACE2 Imaging Representations

**DOI:** 10.1007/s10278-023-00895-w

**Published:** 2023-08-08

**Authors:** Tian Xia, Xiaohang Fu, Michael Fulham, Yue Wang, Dagan Feng, Jinman Kim

**Affiliations:** 1 School of Computer Science, Faculty of Engineering, The University of Sydney, Sydney, NSW 2006 Australia; 2 Department of Molecular Imaging, Royal Prince Alfred Hospital, Camperdown, NSW 2050 Australia; 3 Department of Electrical and Computer Engineering, Virginia Polytechnic Institute and State University, Arlington, VA 22203 USA

**Keywords:** Radiogenomics, Radiomics, COVID-19, ACE2

## Abstract

Coronavirus disease 2019 (COVID-19) is caused by Severe Acute Respiratory Syndrome Coronavirus 2 which enters the body via the angiotensin-converting enzyme 2 (ACE2) and altering its gene expression. Altered ACE2 plays a crucial role in the pathogenesis of COVID-19. Gene expression profiling, however, is invasive and costly, and is not routinely performed. In contrast, medical imaging such as computed tomography (CT) captures imaging features that depict abnormalities, and it is widely available. Computerized quantification of image features has enabled ‘radiogenomics’, a research discipline that identifies image features that are associated with molecular characteristics. Radiogenomics between ACE2 and COVID-19 has yet to be done primarily due to the lack of ACE2 expression data among COVID-19 patients. Similar to COVID-19, patients with lung adenocarcinoma (LUAD) exhibit altered ACE2 expression and, LUAD data are abundant. We present a radiogenomics framework to derive image features (ACE2-RGF) associated with ACE2 expression data from LUAD. The ACE2-RGF was then used as a surrogate biomarker for ACE2 expression. We adopted conventional feature selection techniques including ElasticNet and LASSO. Our results show that: i) the ACE2-RGF encoded a distinct collection of image features when compared to conventional techniques, ii) the ACE2-RGF can classify COVID-19 from normal subjects with a comparable performance to conventional feature selection techniques with an AUC of 0.92, iii) ACE2-RGF can effectively identify patients with critical illness with an AUC of 0.85. These findings provide unique insights for automated COVID-19 analysis and future research.

## Introduction

Coronavirus disease 2019 (COVID-19) caused by the Severe Acute Respiratory Syndrome Coronavirus 2 (SARS-CoV-2) has claimed over 6.5 million lives in more than 200 nations as at October 2022. The clinical manifestations of severe COVID-19 are dominated by respiratory symptoms including acute respiratory distress syndrome (ARDS) [1] and pneumonia, while some patients have also developed severe myocardial damage [2]. Currently, COVID-19 is diagnosed through polymerase chain reaction (PCR) tests and rapid antigen tests to determine the presence of SARS-CoV-2 virus in biological sample [3]. SARS-CoV-2 gains entry to the human body via angiotensin-converting enzyme 2 (ACE2), a membrane-bound aminopeptidase that is abundantly expressed in the lungs and the heart [4, 5]. ACE2 plays a central role in the renin–angiotensin–aldosterone system (RAAS) [6], which has principal effectors that regulate vasoconstriction, oxidative stress, and inflammation [7, 8]. Recent research has associated the pathophysiology of COVID-19 with altered expression of the ACE2 gene after viral infection. Gheware et al. [9] observed markedly increased ACE2 protein expression in lung tissue of patients with severe COVID-19. Other studies analysed the involvement of ACE2 in SARS-CoV and extrapolated to COVID-19, given that SARS-CoV and SARS-CoV2 are genetically similar and induce similar symptomatology [10, 11]. Li et al. [12] found that SARS-CoV2 affects ACE2 expression during viral entry, which may involve local immune responses and result in lung and cardiovascular injury. Similar findings were reported by Tay et al. [13], where SARS-CoV2 infection altered ACE2 expression and resulted in the dysfunction of the RAAS system. RAAS dysfunction therefore results in increased inflammation and vascular permeability in the airways, and acute lung damage. Patients with severe COVID-19 may develop the acute respiratory distress syndrome (ARDS) which can be fatal.

Patients with lung adenocarcinomas (LUAD) also display variable expressions of ACE2 across the different cell types within the tumors [14–16].Similar to COVID-19 infections, altered ACE2 expression in LUAD is associated with the inflammatory signalling pathway via the actions of RAAS [17, 18]. Yang et al. [14] showed the prognostic value of altered ACE2 expression for LUAD, where ACE2 is associated with tumour immune infiltration and prognosis. In addition, Feng et al. [19] has identified ACE2 as an inhibitor of cancer development, metastasis, and angiogenesis in adenocarcinoma-dominated non-small cell lung cancer (NSCLC). Therefore, clinical symptoms of altered ACE2 expression, such as inflammation and ARDS, are comparable in LUAD and COVID-19 [20]. However, gene expression profiling necessitates adequate tissue samples, which are obtained by core biopsies, which capture only a portion of the abnormality, and are invasive and expensive. Thus, gene expression profiling is not routinely done for COVID-19 and, to the best of our knowledge, has not been conducted on large patient cohorts.

Medical imaging, on the other hand, plays a vital role in routine clinical practice for its ability to capture visual representations of the function of organs or tissues (physiology). These visual representations are known as ‘image features’ and they can describe the size and location of abnormalities. Computed tomography (CT) provides an alternate means of detecting COVID-19 by detecting its clinical manifestations in the lung, such as widespread regions of ground glass changes and consolidation [21]. Advances in computerized medical image analysis have enabled ‘radiomics’, a high-throughput and quantitative technique which extracts imaging visual characteristics that cannot be quantified by visual inspection alone [22]. In a recent study, Li and Xia [23] determined the diagnostic value of CT radiomics features for COVID-19. COVID-19 was found to be associated with CT radiomics features such as ground-glass opacities (GGOs), consolidation with vascular enlargement, interlobular and septal thickening.

The diagnostic capabilities of CT enable ‘radiogenomics’, a developing research discipline that aims to identify image features that share statistical associations with molecular characteristics (‘radiogenomics features’). These features can be determined by identifying image features that have statistically significant associations with gene expression [22, 24, 25]. Previous studies have demonstrated that radiogenomics features can detect a variety of diseases other than COVID-19 and predict prognosis and treatment response. An et al. [26] reported that radiogenomics features are associated with Mammalian target of rapamycin (mTOR) pathway gene activity in hepatocellular carcinoma (HCC), where the mTOR signalling pathway governs cellular activities and offers opportunities for targeted anti-tumour treatment. Lee et al. [27] identified a collection of radiogenomics features that are predictive of postsurgical metastases in patients with pathological stage T1 renal cell carcinoma (pT1 RCC). In contrast to conventional imaging features, radiogenomics features have been shown to provide unique insights into intratumor heterogeneity, which can be linked to clinical outcome. Despite the potential of radiogenomics, the association between ACE2 expression and COVID-19 clinical manifestations has not been previously investigated.

In this study, we propose a radiogenomics framework for identifying and selecting radiogenomics features that signify altered ACE2 expressions (‘ACE2-RGF’). This is achieved through the determination of radiogenomics relationships using imaging and ACE2 expression data from LUAD patients. We hypothesize that CT data may be used to derive ACE2-RGF that can serve as surrogate biomarkers for altered ACE2 expression. In addition, it is anticipated that the ACE2-RGF could encode unique insights about pathophysiologic information common to LUAD and COVID-19 and may serve as a biomarker for COVID-19 classification and the identification of critical illness. We investigated our hypotheses on several publicly available CT datasets of lung cancer (LUAD) and COVID-19, and its ability to separate LUAD and COVID-19 from healthy normal patients (hereby denote as ‘normal’), and to identify COVID-19 critical illness from those with mild symptoms.

## Methods

### Materials

We compiled CT scans from multiple public datasets. For LUAD, we used 3 datasets from The Cancer Imaging Archive (TCIA) [28]: i) NSCLC Radiogenomics from Stanford University [29] (‘NRG-S’), ii) NSCLC Radiomics-Genomics from Harvard University [30], (‘NRG-H’), and, iii) NSCLC Radiomics from Harvard University [30] (‘NR-H’). Only NSCLC patients with the LUAD subtype were included. The NRG-S dataset contained scans from 161 patients, 112 also had lung tumour segmentation and 49 had valid ACE2 expression data. The gene expression data were generated with RNA-Seq. The NRG-H dataset comprised CT and gene expression values generated using microarray from 42 patients. There were no corresponding segmentations in the original dataset. We obtained tumour segmentations for NRG-H dataset from an experienced medical imaging specialist (M.F., with > 20 years experience), slice-by-slice, on trans-axial image slices. In addition, the NR-H dataset comprises of 51 CT scans from LUAD patients. There was no corresponding segmentation in the NR-H dataset. In total, there were 254 LUAD CT scans; 91 also had tumour segmentations and ACE2 expression data. One patient from the NRG-S dataset was later removed from our study due to an exceptionally high ACE2 expression level. For examples of COVID-19 and COVID-19-free subjects (normal) patients, we used images from the China Consortium of Chest CT Image Investigation (CC-CCII) [31]. We downloaded all available data and 1,496 COVID-19 and 725 normal scans were studied.

### Experimental Overview

**Fig. 1:**
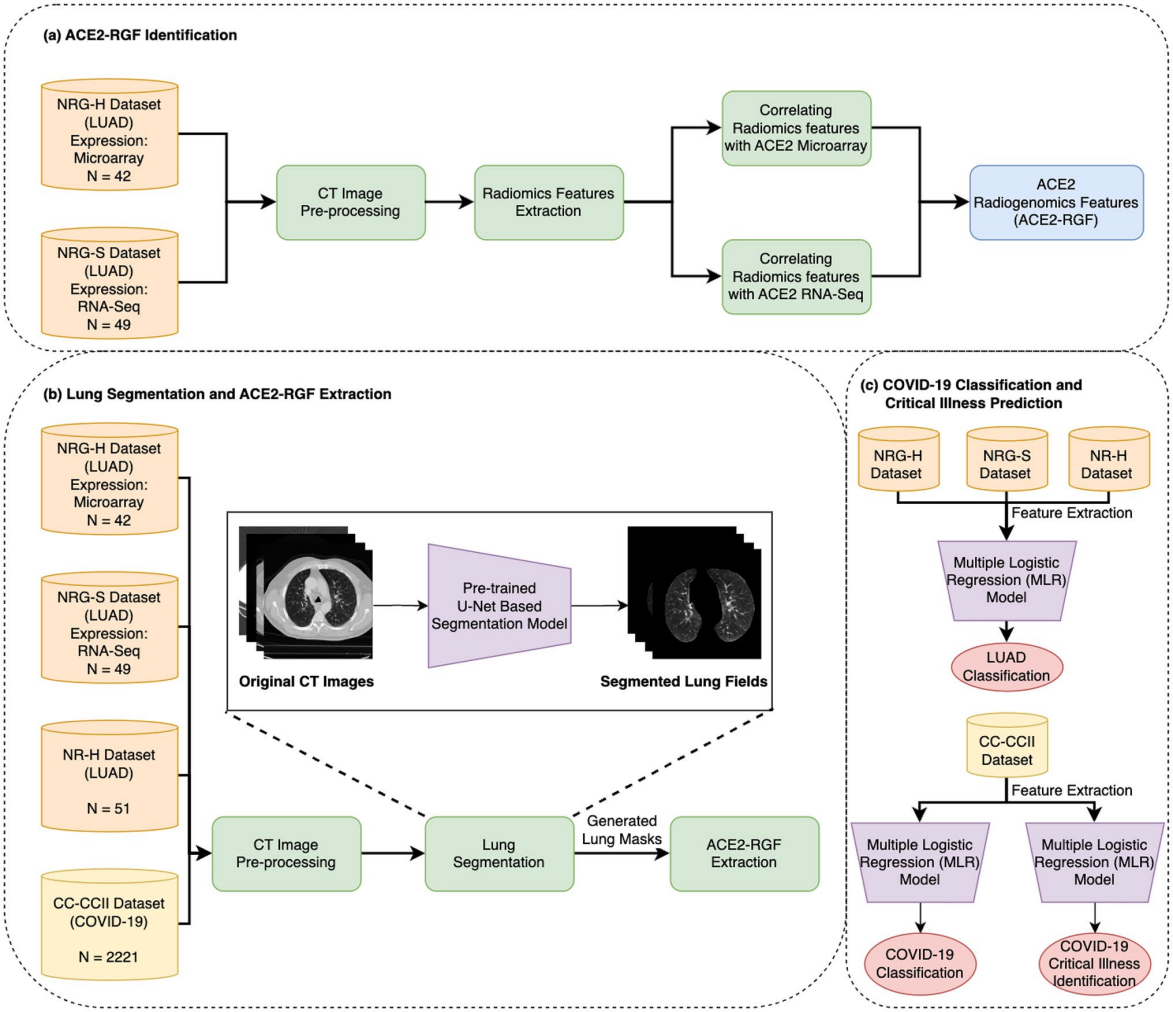
Our proposed radiogenomics framework. It quantifies and identifies ACE2-RGF to construct multiple logistic regression models for classifying COVID-19 from normal subjects and identify critical illness from mild symptoms

In our framework, image features were extracted from the CT. The ACE2-RGF was determined by using Spearman rank correlation between ACE2 expressions and image features from the NRG-S and NRG-H datasets. ACE2-RGF was used to train a multiple logistic regression (MLR) classifier, which comprised a set of coefficients, and two output predictions corresponding to each class (e.g., COVID-19 and normal). The MLR classifiers were trained using LUAD images and were evaluated for their performance for COVID-19 classification and critical illness identification. An overview of our framework is outlined in Fig. 1.

### Image Pre-Processing and Lung Segmentation

All images were converted to Hounsfield units (HU) prior to segmentation and further processing. For the LUAD images, thresholding with a range of [-1,024, 300] HU was applied to be consistent with the CC-CCII dataset. We used an automated lung segmentation algorithm with a pre-trained model [32] to segment the lung regions. This method was based on U-Net and trained on various large CT datasets, including some with COVID-19 examples. Image slices containing fewer than 40% of the greatest number of positively identified lung pixels in any slice of a volume were removed. All slices in an image volume were cropped using the bounding box computed from the sum of the segmentation results (masks) in axial view and then resized to 256 × 256.

### Radiomics Feature Extraction and Correlation Analysis

We extracted image features using the widely applied *pyradiomics* [33] Python package from the tumor regions of the images from the NRG-S and the NRG-H datasets, and from the segmented lung regions of all the available scans. A total of 1,288 features relating to shape, first order statistics, and texture were computed per scan volume. Features were extracted from the original images, derived images using Laplacian of Gaussian (LoG) filtering with 5 different sigma levels, and Wavelet decomposition with different combinations of low (denote as ‘L’) and high-pass (denote as ‘H’) filters on the X, Y and Z dimensions of the image. Shape features were computed only on the original inputs while all other features were extracted from the original and the derivatives. Shape characteristics included volume, surface area, and length. First order statistics, such as mean, kurtosis, and skewness, described the image intensity histogram. Texture features were quantified by means of grey level cooccurrence matrix (GLCM), grey level run length matrix (GLRLM), grey level size zone matrix (GLSZM), neighboring grey tone difference matrix (NGTDM), and grey level dependence matrix (GLDM). GLCM [34] describes the spatial relationship between pixels of similar intensities. GLRLM [35] quantifies the length of consecutive pixels with the same intensity. GLSZM [36] depicts texture homogeneity and areas with the same grey-level. NGTDM [37] quantifies the difference between a pixel and its average neighboring intensities. GLDM [38] represents the connectedness of similar grey-levels.

The extracted image features were subsequently associated with the expression of the ACE2 gene through the utilization of Spearman's rank correlation method, as expressed by the following equation:

1 $$R\left(I, E\right)=1- \frac{6\sum {d}^{2}}{n({n}^{2}-1)}$$

Here, the correlation coefficient *R* represents the relationship between the image features *I* and the ACE2 expression *E*, and it is determined by the differences (*d*) between the ranked values of *I* and *E*. The value of *n* represents the total number of patients included in the analysis. Their significance and stability were evaluated across the NRG-S and NRG-H datasets. Image features that displayed a significant correlation (*p* < 0.05) with ACE2 expression in both datasets were chosen to constitute the ACE2-RGF.

For our framework, Multiple Logistic Regression (MLR) classifiers were used to predict the class (LUAD/COVID-19 or normal) from a CT scan. MLR is a widely used statistical algorithm for modelling the relationship between categorical dependent variables and multiple independent variables [39]. MLR was selected as the classifier over other available classifiers such as Support Vector Machines (SVM). This decision was influenced by MLR's wide utilization in radiogenomics studies [40], owing to its notable interpretability [41]. The classifier comprised of a set of coefficients and two output predictions corresponding to each class.

## Experiments

The proposed radiogenomics framework was assessed by conducting two sets of experiments: i) ACE2-RGF classifying LUAD/normal and COVID-19/normal and, ii) ACE2-RGF classifying COVID-19/normal subjects, and in identifying critical illness subjects.

First, we derived ACE2-RGF from the NRG-S and NRG-H datasets according to their correlation to ACE2 gene profiles; these features were then used with MLR to measure their ability to classify LUAD/normal and COVID-19/normal subjects. Radiomics features were also extracted from the NRG-S and the NRG-H datasets. A variety of conventional feature selection techniques were employed to determine the best representative features for the tasks, including analysis of variance (ANOVA), mutual information [42], recursive feature elimination (RFE) [43] using a support vector classifier estimator, minimum redundancy maximum relevance (mRMR) [44], ReliefF [45], random forest with 100 estimators and Gini impurity, least absolute shrinkage and selection operator (Lasso) [46], Ridge, and Elastic Net [47] with an L1 ratio of 0.5. These conventional feature selection techniques were implemented with their default parameters to ensure model generalizability and reproducibility. Our approach aligns with recent radiomics and radiogenomics machine learning research [48, 49]. The resulting collections of selected image features are denoted as LUAD-RF. For instance, LUAD-RF_*ANOVA*_ represents radiomics features extracted from LUAD subjects and was processed using the ANOVA feature selection technique. The performance of ACE2-RGF was compared to LUAD-RF and all extracted radiomics features (‘LUAD-AF’).

Next, the ACE2-RGF was used with MLR to measure its ability to separate COVID-19/normal. For this experiment, radiomics features were extracted from CC-CCII datasets. The same feature selection techniques were applied to the extracted radiomics features and the resulting collection of selected image features were denoted as COVID-19-RF. The performance of ACE2-RGF was compared to COVID-19-RF and all extracted radiomics features (‘COVID-19-AF’).

Lastly, our ACE2-RGF was used with MLR to measure its ability for identifying COVID-19 critical illness. For this experiment, radiomics features were also extracted from CC-CCII datasets. We followed the same feature selection procedure as for the extracted radiomics features and the resulting collection of selected image features were denoted as COVID-Crt-RF. The performance of ACE2-RGF was compared to COVID-Crt-RF and all extracted radiomics features (‘COVID-Crt-AF’).

fivefold cross-validation was performed for all experiments. We randomly sampled 250 patients each of LUAD and normal classes (500 in total), and further randomly divided the sample into training and validation sets with an 80/20 split, resulting in 200 examples for training and 50 for validation from each class. Identical patient splits were used for both methods and no subject existed in both the training and validation sets of a fold. For the test set, all available COVID-19 patients and control subjects not chosen in the cross-validation sample were included. Each training set, despite having different datasets to each other, extracted the same set of ACE2-RGF features. We evaluated our MLR models using performance metrics including accuracy (ACC), area under the ROC curve (AUC), F_1_ score, F_1_ score of only the positive (LUAD/COVID-19) class (F_1_ POS), precision (PREC), recall (RECA), and specificity (SPEC). We define the best model based on the highest average score between F_1_ and AUC on the validation set of its fold.

## Results

### ACE2-RGF for Classifying LUAD, COVID-19, and Normal Subjects

The ACE2-RGF had 12 features that were significantly correlated with the expression of the ACE2 gene (Table 1). These features were derived from the GLCM, GLRLM, GLSZM, and GLDM, which are all descriptors of image texture. Eight of the 12 features related to textural "emphasis," which describes the proportion of various grey-level values and zones of varied sizes within an image. Notably, all 12 image features were extracted from the derived images using LoG filtering with sigma levels of 3 and 4.

**Table 1:** ACE2-RGF image features (12 features)

**Feature Name**	**Origin**
log_sigma_3_0_mm_3D_glcm_Autocorrelation	GLCM
log_sigma_3_0_mm_3D_glcm_JointAverage	GLCM
log_sigma_3_0_mm_3D_glrlm_HighGrayLevelRunEmphasis	GLRLM
log_sigma_3_0_mm_3D_gldm_HighGrayLevelEmphasis	GLSZM
log_sigma_3_0_mm_3D_gldm_LargeDependenceLowGrayLevelEmphasis	GLSZM
log_sigma_4_0_mm_3D_glcm_Autocorrelation	GLCM
log_sigma_4_0_mm_3D_glcm_JointAverage	GLCM
log_sigma_4_0_mm_3D_glrlm_LongRunLowGrayLevelEmphasis	GLSZM
log_sigma_4_0_mm_3D_glrlm_LowGrayLevelRunEmphasis	GLRLM
log_sigma_4_0_mm_3D_gldm_HighGrayLevelEmphasis	GLSZM
log_sigma_4_0_mm_3D_gldm_LargeDependenceLowGrayLevelEmphasis	GLSZM
log_sigma_4_0_mm_3D_gldm_LowGrayLevelEmphasis	GLRLM

Tables 2 and 3 compare the performance for LUAD-AF, ACE2-RGF, and LUAD-RF for classifying LUAD from normal subjects and classifying COVID-19 from normal subjects. LUAD-AF and LUAD-RF demonstrated superior performance than ACE2-RGF for classifying LUAD from normal patients. However, MLR classifiers showed substantial decreases in performance when LUAD-AF and LUAD-RF were used as inputs for COVID-19 classification. In contrast, MLR with ACE2-RGF showed consistent performance for classifying LUAD and COVID-19 from normal subjects.

**Table 2:** Performance of the MLR models for classifying LUAD from normal subjects using i) LUAD with all extracted radiomics features (LUAD-AF), ii) ACE2-RGF, and iii) LUAD with selected radiomics features (LUAD-RF). LUAD Radiomics features were extracted from the NRG-H and NRG-S datasets. ACE2-RGF was derived and extracted from the NRG-H and NR

**Input**	**ACC**	**AUC**	**F**_**1**_	**F**_**1**_** POS**	**PREC**	**RECA**	**SPEC**
**LUAD-AF**	0.99 (± 0.01)	1.00 (± 0.01)	0.99 (± 0.01)	0.99 (± 0.01)	0.99 (± 0.02)	0.99 (± 0.01)	0.99 (± 0.02)
**ACE2-RGF**	0.85 (± 0.02)	0.91 (± 0.03)	0.85 (± 0.02)	0.87 (± 0.02)	0.79 (± 0.02)	0.95 (± 0.02)	0.75 (± 0.03)
**LUAD-RF**_**ANOVA**_	0.96 (± 0.01)	1.00 (± 0.00)	0.96 (± 0.01)	0.96 (± 0.01)	0.95 (± 0.02)	0.97 (± 0.02)	0.95 (± 0.02)
**LUAD-RF**_**Mutual Info**_	0.97 (± 0.01)	1.00 (± 0.00)	0.97 (± 0.01)	0.97 (± 0.01)	0.97 (± 0.02)	0.98 (± 0.01)	0.97 (± 0.02)
**LUAD-RF**_**RFE**_	1.00 (± 0.01)	1.00 (± 0.00)	1.00 (± 0.01)	1.00 (± 0.01)	1.00 (± 0.01)	1.00 (± 0.01)	1.00 (± 0.01)
**LUAD-RF**_**mRMR**_	0.98 (± 0.01)	1.00 (± 0.00)	0.98 (± 0.01)	0.98 (± 0.01)	0.98 (± 0.02)	0.98 (± 0.01)	0.98 (± 0.02)
**LUAD-RF**_**ReliefF**_	0.92 (± 0.02)	0.97 (± 0.01)	0.92 (± 0.03)	0.92 (± 0.03)	0.91 (± 0.03)	0.93 (± 0.05)	0.91 (± 0.04)
**LUAD-RF**_**Forest**_	0.97 (± 0.02)	1.00 (± 0.00)	0.97 (± 0.02)	0.97 (± 0.02)	0.98 (± 0.02)	0.97 (± 0.02)	0.98 (± 0.02)
**LUAD-RF**_**LASSO**_	0.99 (± 0.01)	1.00 (± 0.00)	0.99 (± 0.01)	0.99 (± 0.01)	1.00 (± 0.01)	0.99 (± 0.01)	1.00 (± 0.01)
**LUAD-RF**_**Ridge**_	0.99 (± 0.01)	1.00 (± 0.00)	0.99 (± 0.01)	0.99 (± 0.01)	0.99 (± 0.02)	1.00 (± 0.01)	0.99 (± 0.02)
**LUAD-RF**_**Elastic Net**_	0.99 (± 0.01)	1.00 (± 0.00)	0.99 (± 0.01)	0.99 (± 0.01)	0.99 (± 0.02)	0.99 (± 0.01)	0.99 (± 0.02)

**Table 3:** Performance of the MLR models for classifying COVID-19 from normal subject using i) LUAD-AF, ii) ACE2-RGF, and iii) LUAD-RF. Radiomics features were extracted from the NRG-S and NRG-H datasets. ACE2-RGF was derived and extracted from the NRG-H and NRG-S datasets

**Input**	**ACC**	**AUC**	**F**_**1**_	**F**_**1**_** POS**	**PREC**	**RECA**	**SPEC**
**LUAD-AF**	0.28 (± 0.01)	0.70 (± 0.05)	0.25 (± 0.01)	0.09 (± 0.01)	0.99 (± 0.01)	0.05 (± 0.01)	1.00 (± 0.00)
**ACE2-RGF**	0.85 (± 0.02)	0.83 (± 0.01)	0.80 (± 0.02)	0.90 (± 0.01)	0.91 (± 0.00)	0.89 (± 0.03)	0.72 (± 0.02)
**LUAD-RF**_**ANOVA**_	0.37 (± 0.03)	0.82 (± 0.03)	0.37 (± 0.03)	0.31 (± 0.05)	0.91 (± 0.02)	0.19 (± 0.03)	0.94 (± 0.01)
**LUAD-RF**_**Mutual Info**_	0.38 (± 0.07)	0.83 (± 0.01)	0.37 (± 0.07)	0.31 (± 0.11)	0.90 (± 0.02)	0.20 (± 0.09)	0.94 (± 0.01)
**LUAD-RF**_**RFE**_	0.28 (± 0.01)	0.52 (± 0.09)	0.24 (± 0.01)	0.09 (± 0.02)	1.00 (± 0.00)	0.04 (± 0.01)	1.00 (± 0.00)
**LUAD-RF**_**mRMR**_	0.28 (± 0.02)	0.64 (± 0.05)	0.25 (± 0.03)	0.11 (± 0.05)	0.86 (± 0.05)	0.06 (± 0.03)	0.97 (± 0.01)
**LUAD-RF**_**ReliefF**_	0.65 (± 0.03)	0.81 (± 0.03)	0.63 (± 0.03)	0.70 (± 0.04)	0.96 (± 0.01)	0.56 (± 0.05)	0.92 (± 0.02)
**LUAD-RF**_**Forest**_	0.34 (± 0.02)	0.87 (± 0.01)	0.33 (± 0.03)	0.24 (± 0.05)	0.92 (± 0.01)	0.14 (± 0.04)	0.96 (± 0.01)
**LUAD-RF**_**LASSO**_	0.30 (± 0.01)	0.55 (± 0.04)	0.28 (± 0.02)	0.15 (± 0.03)	1.00 (± 0.00)	0.08 (± 0.02)	1.00 (± 0.00)
**LUAD-RF**_**Ridge**_	0.27 (± 0.00)	0.61 (± 0.03)	0.23 (± 0.00)	0.07 (± 0.01)	1.00 (± 0.01)	0.03 (± 0.00)	1.00 (± 0.00)
**LUAD-RF**_**Elastic Net**_	0.28 (± 0.01)	0.69 (± 0.06)	0.24 (± 0.01)	0.09 (± 0.01)	0.98 (± 0.01)	0.05 (± 0.01)	1.00 (± 0.00)

### MLR for COVID-19 Classification

For COVID-19 classification, radiomics features that were frequently selected by conventional feature selection techniques (Table 4) were exclusively derived from decomposed images using 3D wavelet decomposition with LLH filters. Notably, none of these wavelet features overlap to ACE2-RGF.

**Table 4:** Top 12 radiomics features that were frequently selected by conventional image feature selection techniques for COVID-19 classification

Feature Name	*Frequency of Selection (%)*
wavelet_LLH_firstorder_Maximum	60.0
wavelet_LLH_firstorder_Range	54.3
wavelet_LLH_glszm_HighGrayLevelZoneEmphasis	48.6
wavelet_LLH_glrlm_HighGrayLevelRunEmphasis	45.7
wavelet_LLH_glszm_SmallAreaHighGrayLevelEmphasis	42.9
wavelet_LLH_glcm_Autocorrelation	42.9
wavelet_LLH_gldm_HighGrayLevelEmphasis	40.0
wavelet_LHH_firstorder_Mean	37.1
wavelet_LLH_glrlm_ShortRunHighGrayLevelEmphasis	37.1
wavelet_LLH_gldm_SmallDependenceHighGrayLevelEmphasis	31.4
wavelet_LLH_glszm_GrayLevelVariance	31.4
wavelet_LLH_glrlm_LongRunHighGrayLevelEmphasis	31.4

Table 5 presents the performance for COVID-19-AF, ACE2-RGF, and COVID-19-RF for classifying COVID-19 from normal subjects. Although ACE2-RGF did not achieve the highest performance for classifying COVID-19, the ACE2-RGF performed comparably or better in AUC, F_1 _POS, accuracy, and recall when compared to a variety of COVID-19-RF. Upon fusing ACE2-RGF with COVID-19-RF, the resulting feature set comprised a total of 24 features, with 12 from each. The utilization of the combined feature set lead to improved performance in several MLR models for COVID-19 classification (Table 6). Notably, among the MLR models with improved performance, ACE2-RGF typically improved the F_1_, F_1_POS, and precision of those models.

**Table 5:** Performance of the MLR models for classifying COVID-19 from normal subject using i) COVID-19 with all extracted radiomics features (COVID-19-AF), ii) ACE2-RGF, and iii) COVID-19 with selected radiomics features (COVID-19-RF). COVID-19-AF Radiomics features were extracted from CT images of the CC-CCII dataset. ACE2-RGF was derived from the NRG-H and NRG-S datasets and was extracted from the CC-CCII dataset

**Input**	**ACC**	**AUC**	**F**_**1**_	**F**_**1**_** POS**	**PREC**	**RECA**	**SPEC**
**COVID-19-AF**	0.94 (± 0.02)	0.99 (± 0.01)	0.94 (± 0.02)	0.94 (± 0.02)	0.97 (± 0.01)	0.91 (± 0.04)	0.97 (± 0.01)
**ACE2-RGF**	0.82 (± 0.05)	0.92 (± 0.03)	0.82 (± 0.05)	0.83 (± 0.04)	0.79 (± 0.04)	0.87 (± 0.05)	0.77 (± 0.05)
**COVID-19-RF**_**ANOVA**_	0.89 (± 0.02)	0.94 (± 0.01)	0.89 (± 0.02)	0.88 (± 0.02)	0.90 (± 0.03)	0.87 (± 0.02)	0.90 (± 0.03)
**COVID-19-RF**_**Mutual Info**_	0.88 (± 0.04)	0.95 (± 0.02)	0.88 (± 0.04)	0.88 (± 0.04)	0.89 (± 0.05)	0.88 (± 0.03)	0.89 (± 0.06)
**COVID-19-RF**_**RFE**_	0.94 (± 0.03)	0.98 (± 0.02)	0.94 (± 0.03)	0.94 (± 0.03)	0.95 (± 0.02)	0.94 (± 0.04)	0.95 (± 0.02)
**COVID-19-RF**_**mRMR**_	0.91 (± 0.01)	0.96 (± 0.01)	0.91 (± 0.01)	0.91 (± 0.01)	0.93 (± 0.02)	0.88 (± 0.02)	0.93 (± 0.02)
**COVID-19-RF**_**ReliefF**_	0.64 (± 0.02)	0.69 (± 0.04)	0.63 (± 0.03)	0.64 (± 0.04)	0.63 (± 0.02)	0.66 (± 0.08)	0.61 (± 0.06)
**COVID-19-RF**_**Forest**_	0.92 (± 0.03)	0.97 (± 0.01)	0.92 (± 0.03)	0.92 (± 0.03)	0.94 (± 0.03)	0.91 (± 0.04)	0.94 (± 0.03)
**COVID-19-RF**_**LASSO**_	0.96 (± 0.01)	0.99 (± 0.01)	0.96 (± 0.01)	0.96 (± 0.01)	0.98 (± 0.01)	0.95 (± 0.02)	0.98 (± 0.01)
**COVID-19-RF**_**Ridge**_	0.96 (± 0.02)	0.99 (± 0.01)	0.96 (± 0.02)	0.96 (± 0.02)	0.98 (± 0.02)	0.94 (± 0.03)	0.98 (± 0.02)
**COVID-19-RF**_**Elastic Net**_	0.94 (± 0.02)	0.98 (± 0.01)	0.94 (± 0.02)	0.93 (± 0.02)	0.96 (± 0.02)	0.91 (± 0.04)	0.96 (± 0.02)

**Table 6:** Performance of MLR models for classifying COVID-19 subject from normal subjects. ACE2-RGF was fused with COVID-19-RF. COVID-19-RF Radiomics features were extracted from the CC-CCII dataset. ACE2-RGF was derived from the NRG-H and NRG-S datasets and was extracted from the CC-CCII dataset

**Input**	**ACC**	**AUC**	**F**_**1**_	**F**_**1**_** POS**	**PREC**	**RECA**	**SPEC**
**COVID-19-RF**_**ANOVA**_** + ACE2-RGF**	0.88 (± 0.02)	**0.95** (± 0.02)	0.88 (± 0.02)	0.88 (± 0.02)	0.89 (± 0.03)	**0.88** (± 0.02)	0.89 (± 0.03)
**COVID-19-RF**_**Mutual Info**_** + ACE2-RGF**	**0.90** (± 0.03)	0.95 (± 0.01)	**0.90** (± 0.03)	**0.89** (± 0.03)	**0.91** (± 0.03)	0.88 (± 0.04)	**0.91** (± 0.04)
**COVID-19-RF**_**RFE**_** + ACE2-RGF**	**0.95** (± 0.03)	0.98 (± 0.01)	**0.95** (± 0.03)	**0.95** (± 0.03)	**0.96** (± 0.02)	0.94 (± 0.03)	**0.96** (± 0.02)
**COVID-19-RF**_**mRMR**_** + ACE2-RGF**	0.90 (± 0.02)	0.96 (± 0.01)	0.90 (± 0.02)	0.90 (± 0.02)	0.92 (± 0.01)	0.87 (± 0.03)	0.92 (± 0.01)
**COVID-19-RF**_**ReliefF**_** + ACE2-RGF**	**0.91** (± 0.02)	**0.96** (± 0.01)	**0.91** (± 0.02)	**0.91** (± 0.02)	**0.92** (± 0.02)	**0.90** (± 0.05)	**0.92** (± 0.03)
**COVID-19-RF**_**Forest**_** + ACE2-RGF**	0.86 (± 0.03)	0.93 (± 0.01)	0.86 (± 0.03)	0.86 (± 0.03)	0.86 (± 0.04)	0.86 (± 0.04)	0.86 (± 0.04)

Numbers in bold indicate improved performance from fusing ACE2-RGF with COVID-19-RF

### MLR for COVID-19 Critical Illness Identification

For COVID-19 critical illness identification, image features commonly selected using conventional feature selection techniques (Table 7) were derived from log and wavelet filters. Notably, none of these wavelet features overlapped ACE2-RGF. Table 8 presents the performance for COVID-Crt-AF, ACE2-RGF, and COVID-Crt-RF for identifying COVID-19 critical illness. Although ACE2-RGF did not achieve the greatest performance for COVID-19 critical illness identification, the gap between the top performing models and ACE2-RGF was within 5% in AUC.

**Table 7:** Top 12 radiomics features that were frequently selected by conventional image feature selection techniques for COVID-19 critical illness identification

Feature Name	*Frequency of Selection (%)*
wavelet_LLL_glcm_Correlation	42.9
wavelet_HLH_glcm_Idn	37.1
wavelet_HLL_firstorder_Kurtosis	28.6
wavelet_LLL_glcm_Idmn	22.9
wavelet_LLH_gldm_SmallDependenceLowGrayLevelEmphasis	20.0
wavelet_HLH_glcm_Idmn	17.1
wavelet_HLH_firstorder_Kurtosis	17.1
wavelet_LLH_glcm_JointAverage	17.1
log_sigma_4_0_mm_3D_gldm_SmallDependenceEmphasis	17.1
log_sigma_1_0_mm_3D_glcm_Idmn	17.1
wavelet_LLL_glcm_Imc2	17.1
wavelet_HLL_glcm_Idn	14.3

**Table 8:** Performance of MLR models for identifying COVID-19 critical illness using various feature selection methods. COVID-Crt-AF radiomics features were extracted from the CC-CCII dataset. ACE2-RGF was derived from the NRG-H and NRG-S datasets and was extracted from the CC-CCII dataset

**Input**	**ACC**	**AUC**	**F**_**1**_	**F**_**1**_** POS**	**PREC**	**RECA**	**SPEC**
**COVID-Crt-AF**	0.81 (± 0.03)	0.88 (± 0.02)	0.80 (± 0.04)	0.78 (± 0.05)	0.75 (± 0.06)	0.82 (± 0.05)	0.80 (± 0.06)
**ACE2-RGF**	0.77 (± 0.02)	0.85 (± 0.02)	0.77 (± 0.02)	0.73 (± 0.04)	0.73 (± 0.07)	0.74 (± 0.08)	0.80 (± 0.04)
**COVID-Crt-RF**_**ANOVA**_	0.81 (± 0.02)	0.89 (± 0.02)	0.80 (± 0.02)	0.76 (± 0.03)	0.80 (± 0.05)	0.73 (± 0.07)	0.87 (± 0.03)
**COVID-Crt-RF**_**Mutual Info**_	0.81 (± 0.03)	0.88 (± 0.02)	0.80 (± 0.03)	0.76 (± 0.04)	0.79 (± 0.05)	0.74 (± 0.08)	0.86 (± 0.03)
**COVID-Crt-RF**_**RFE**_	0.80 (± 0.05)	0.88 (± 0.03)	0.79 (± 0.06)	0.75 (± 0.08)	0.77 (± 0.08)	0.73 (± 0.11)	0.84 (± 0.05)
**COVID-Crt-RF**_**mRMR**_	0.84 (± 0.02)	0.89 (± 0.02)	0.84 (± 0.02)	0.81 (± 0.03)	0.84 (± 0.05)	0.78 (± 0.05)	0.89 (± 0.03)
**COVID-Crt-RF**_**ReliefF**_	0.48 (± 0.04)	0.46 (± 0.06)	0.45 (± 0.05)	0.34 (± 0.10)	0.38 (± 0.09)	0.33 (± 0.13)	0.60 (± 0.11)
**COVID-Crt-RF**_**Forest**_	0.79 (± 0.02)	0.86 (± 0.02)	0.79 (± 0.03)	0.75 (± 0.05)	0.78 (± 0.06)	0.73 (± 0.12)	0.84 (± 0.05)
**COVID-Crt-RF**_**LASSO**_	0.79 (± 0.04)	0.87 (± 0.02)	0.79 (± 0.04)	0.76 (± 0.06)	0.75 (± 0.06)	0.77 (± 0.08)	0.81 (± 0.05)
**COVID-Crt-RF**_**Ridge**_	0.77 (± 0.06)	0.84 (± 0.04)	0.76 (± 0.06)	0.73 (± 0.07)	0.73 (± 0.08)	0.73 (± 0.22)	0.79 (± 0.09)
**COVID-Crt-RF**_**Elastic Net**_	0.81 (± 0.04)	0.89 (± 0.02)	0.81 (± 0.05)	0.79 (± 0.06)	0.76 (± 0.08)	0.82 (± 0.05)	0.81 (± 0.07)

## Discussion

Our main findings are that our framework can: i) encode ACE2-RGF imaging biomarkers using LUAD data, which are distinct to radiomics features extracted for COVID-19 classification and critical illness identification; ii) the ACE2-RGF can distinguish COVID-19 from normal subjects, and can be combined with COVID-19 RF to improve classification performance; iii) the ACE2-RGF can also effectively identify COVID-19 patients with critical illness and, iv) the ACE2-RGF can be used as a biomarker for various applications, as shown for both COVID-19 classification and critical illness identification.

The ACE2-RGF comprises 12 radiomics features (Table 1) that encodes textural information in CT images. Notably, none of the ACE2-RGF features were among the most frequently selected features when compared with COVID-19-RF (Table 4) and COVID-Crt-RF (Table 7). The ACE2-RGF encoded texture descriptors are a 2D isotropic quantification of the second spatial derivative of an image, and they identify locations with rapid intensity changes within the CT image. Such ACE2-RGF encoded textural information were consistent to the CT findings reported in ARDS and COVID-19 [50, 51], including ground glass opacity, vascular enlargement and crazy-paving pattern. In contrast, the COVID-19-RF encoded statistical and texture features from decomposed images using 3D wavelet decomposition with LLH filters. In comparison, COVID-Crt-RF encoded a distinct collection of image features that were derived from decomposed images using a variety of low and high-pass filters, including LLL, LLH, HLL, and HLH filters and LoG filtered image with Gaussian sigma values at 1 and 4 mm. Our findings indicate that our radiogenomics framework enabled the derivation of image features associated with ACE2 and encoded unique features regarding disease manifestation related to variations in ACE2 expression. In contrast, conventional machine learning-based approaches quantify and select image features that are optimized for particular tasks, thus may neglect important imaging representations related to the pathophysiology of the disease. This is owing to the possibility for multiple ‘optimal’ feature sets to be selected for a particular task, despite different feature sets may offer distinct information [52, 53].

When compared to LUAD-AF and LUAD-RF variants, our radiogenomics framework derived ACE2-RGF demonstrated consistent performance for classifying LUAD (Table 2) and COVID-19 (Table 3) patients from normal subjects. MLR models using LUAD-AF and LUAD-RF demonstrated a substantial decline in performance for classifying COVID-19 patients from normal subjects. Our results show that our framework derived ACE2-RGF encoded imaging representations of pathophysiology information that are common to LUAD and COVID-19. Despite the ACE2-RGF having inferior performance when compared with COVID-19-RF for separating COVID-19 patients from normal subjects (Table 5), the use of ACE2-RGF did not require identifying and extracting COVID-19-RF features. Our findings indicate that the ACE2-RGF encoded imaging representations are associated with alterations in ACE2 expression and are relevant to the pathophysiology of both LUAD and COVID-19. However, such information may not provide the optimal classification value that is specific to both LUAD and COVID-19.

Notably, MLR models trained with COVID-19-AF performed similarly to MLR models trained with multiple COVID-19-RF in classifying COVID-19 patients from healthy subjects (Table 2). Our findings suggest that despite radiomics features (COVID-19-AF) may encode distinctive information, these features have demonstrated their capability to classify COVID-19 when used collectively. In contrast, the conventional machine learning frameworks that quantify task-specific image features may neglect radiomics features that encode relevant information for classifying COVID-19, such as statistical and textural features using various LoG filters.

The classification performance for COVID-19 was enhanced when ACE2-RGF was fused with COVID-19-RF (Table 6). In contrast to COVID-19-RF, ACE2-RGF encoded distinct pathophysiological image features linked with COVID-19, and therefore is complementary to COVID-19-RF. Our results suggest that the conventional machine learning frameworks that quantify task-specific image features may neglect the underlying pathophysiology information of COVID-19 and its clinical manifestation due to altered ACE2 expression. For instance, the involvement of the lower respiratory tract in individuals with early-stage or moderate COVID-19 and the possibility of ARDS progression [54].

Our framework showed it could identify COVID-19 patients with critical illness. The performance of the MLR model trained with ACE2-RGF for identifying COVID-19 critical illness was similarly to that of models trained with COVID-Crt-RF (Table 8). Our findings suggest that the ACE2-RGF may not contain imaging representations exclusive to COVID-19 critical illness status, but rather imaging characteristics associated with ACE2 expression alterations that are tied with the progression of COVID-19 critical illness [55]. Notably, the performance gap between ACE2-RGF and the best performing COVID-Crt-RF for identifying COVID-19 critical illness was less than the gap between ACE2-RGF and the best performing COVID-19-RF for COVID-19 classification. One explanation of our finding is that patients with COVID-19 critical illness commonly have multiple complications that are related or results of ACE2 and RAAS failure, such as ARDS [56, 57].

Our framework demonstrated potential to serve as an imaging biomarker for COVID-19 classification and COVID-19 critical illness identification using the same set of ACE2-RGF. We attribute this to the encoding of altered ACE2 expression in ACE2-RGF. Recent research has implicated the role of ACE2 in the infection, development, and clinical manifestations of COVID in the human body [58]. It is also suggested that ACE2 and its variants affect the binding of SARS-COV2 virus and hence the disease severity following COVID-19 infection [59]. Therefore, our framework has the potential to serve as a valuable biomarker that complements existing image-based frameworks and offer new research possibilities to derive additional features for future automated COVID-19 classification and critical illness identification.

We used traditional handcrafted image features encompassing shape, first-order statistics, and texture. These features are widely adopted for radiogenomics research due to its wide acceptability, comprehension and for its explainability. Recently, deep learning feature extractors have made significant advancements, notably on extracting a complementary set of deep image features to the handcrafted features. For instance, in a recent study by Xia et al. [25] on lung cancer radiogenomics, deep learning features were found to generate unique features that differed from the traditional set. However, these deep learning features lacked interpretability and descriptiveness. In our study, our primary focus was to analyze the ability to encode ACE2-RGF from CT images while providing explanatory insights, which the traditional handcrafted feature set adequately fulfilled. In future work, we plan to explore whether deep learning features can complement our study and offer additional insights.

A limitation of our study is the lack of ACE2 expression for the COVID-19 patients. This limits the ability to optimize the ACE2-RGF for COVID-19 classification and critical illness. We anticipate that with ACE2 expression data of COVID-19 patients, our model can be improved by identifying and selecting ACE2-RGF directly on COVID-19 imaging data. In addition, with the increasing availability of data on COVID-19 critical illness and ACE2 expression, our future work will explore and assess the performance and robustness of the proposed radiogenomics framework across multiple independent datasets.

## Conclusion

We proposed a radiogenomics framework that leverages the potential of CT to capture molecular variations that accompany altered ACE2 expression. Our framework derives ACE2-RGF: a collection of image features that are associated with ACE2 expressions to classify COVID-19 patients. Our proposed framework has potential to serve as an imaging biomarker for COVID-19 classification and COVID-19 critical illness identification using the same set of ACE2-RGF. Our proposed radiogenomics framework can complement existing image-based frameworks and offer new research possibilities that offer additional insights for future automated COVID-19 classification and critical illness identification.

## Data Availability

Publicly available datasets were analyzed in this study. The datasets can be found below: 1. TCIA: https://www.cancerimagingarchive.net, 2. CC-CCII: http://ncov-ai.big.ac.cn/download?lang=en.
